# Leaving no one behind: addressing the sexuality of people with disabilities

**DOI:** 10.1186/s12939-024-02219-y

**Published:** 2024-06-28

**Authors:** Obasanjo Afolabi Bolarinwa, Clifford Odimegwu, Yusuff Adebayo Adebisi

**Affiliations:** 1 Department of Demography and Population Studies, University of Witwatersrand, Johannesburg, South Africa; 2 Department of Public Health, York St John University, London, UK; 3 College of Social Sciences, University of Glasgow, Glasgow, UK

**Keywords:** Sexual health, Sexuality, Non-sexual, People with disability, Leaving no one behind

## Abstract

Addressing the sexuality of individuals with disabilities is important within the framework of global health and societal inclusivity. Despite comprising 16% of the world’s population, this demographic faces sexual autonomy inequality. Acknowledging this intersection is pertinent for achieving inclusive healthcare and upholding the commitments of the 1994 International Conference on Population and Development and the 2006 United Nations Convention on the Rights of Persons with Disabilities. Dispelling stereotypes and promoting dialogue are key to empowering individuals with disabilities and ensuring equitable access to sexual health resources. Integrating sexual health and rights into broader healthcare systems is vital for creating an inclusive society where no one is left behind. This article advocates for the need to address the specific sexual health needs and rights of individuals with disabilities, to implement inclusive policies, and to foster a healthcare environment that respects and supports their autonomy and dignity.

In the context of global health and societal inclusivity, there exists a silent yet important issue that requires collective attention – the sexuality of individuals living with disabilities. Approximately 1.3 billion individuals, or 16% of the world’s population, experience major disability [1]. This equates to one in every six people [1], marking a substantial demographic that necessitates targeted consideration in healthcare planning and policy [2]. While the world strides towards inclusivity, the sexual autonomy and well-being of individuals with disabilities often remain in the shadows and often unacknowledged by the stakeholders. As we acknowledge the three decades of the 1994 International Conference on Population and Development (ICPD), held in Cairo, Egypt [3], which gave birth to the widely agreed platform for promoting and improving sexual and reproductive health and rights for all populations, it is imperative to spotlight this intersection.

Additionally, the 2006 United Nations Convention on the Rights of Persons with Disabilities (CRPD) reinforces the need to ensure that the conversation around sexual health and rights extends to all members of our society as agreed in the conference [4]. In the same vein, a prominent disability organisation names “Missing Billion” in their recent report advocate for the need to reach the unreached to ensure that no one is left behind irrespective of their sexuality, ensuring the need to prioritise the sexual autonomy of people with disabilities [5].

Sexual expression is a vital aspect of human life, yet those with disabilities frequently encounter various obstacles that limit their sexual rights. These include a scarcity of tailored education and healthcare services, as well as the prevalence of detrimental stereotypes and discrimination that undermine their sexual autonomy [6]. Overcoming these societal barriers is essential to create an environment that not only acknowledges but also supports individuals with disabilities, allowing them to make well-informed decisions regarding their sexual health. Additionally, the dearth of research and practical interventions to understand fully the sexuality of persons with disabilities points to an urgent need for focused academic and practical initiatives in this field [7].

Several research outputs by Groce and colleagues have created an understanding of the intersection between people with disability and HIV/AIDS, given a solidifies arguments for future research to ride on [8, 9]. However, discourse around the overall sexuality of people with disability in relation to both ICPD and CRPD agreement in accelerating the achievement of the United Nations’ Sustainable Development Goal 3 by 2030 has received little or no attention [10].

The significance of this discourse cannot be understated, and this is also an important step towards realising the United Nations’ Sustainable Development Goals 3, and in particular, the commitment to leave no one behind [10]. This entails advocating for the rights and dignity of all individuals, ensuring equitable treatment and opportunities for people with disabilities to lead fulfilling lives [11]. Dispelling stereotypes is a vital part of this process. Societal myths often portray individuals with disabilities as either sexually deviant or non-sexual, denying them the respect and recognition of their right to sexual autonomy. Empowering individuals with disabilities begins with an informed dialogue that facilitates their assertion of sexuality and ensures access to essential resources for their sexual well-being. It is within such dialogues that misconceptions can be dispelled, and a path toward equitable resources can be established, allowing for a comprehensive approach to sexuality that is both inclusive and respectful of diversity.

Future research should focus on developing tailored sexual health education programs for individuals with disabilities, formulating inclusive healthcare policies, and studying the impact of societal stereotypes on their sexual autonomy. At the same time, interdisciplinary collaboration among education, healthcare, and social services through workshops and community forums can foster integrated support systems.

Furthermore, hosting policy roundtables with key stakeholders and launching public awareness campaigns will further promote understanding and support for the sexual rights and autonomy of individuals with disabilities. For instance, initiatives such as Sweden’s comprehensive sexual education for people with disabilities [12] and Australia’s National Disability Insurance Scheme (NDIS) [13] effectively address these issues, showing that strong societal commitment can erase barriers and uphold rights for everyone.

As we strive for a society that embodies equity, integrating sexual health and rights within the broader health care and social support systems for individuals with disabilities is vital. We must acknowledge and advocate for the sexuality of people with disabilities, strenghtening the narrative around disabilities to include all aspects of living, especially sexuality, to truly fulfil our commitment to an inclusive world where no one is left behind whilst we continue to uphold the International Conference on Population and Development 1994 and the 2006 CRPD agreements.

## Data Availability

No datasets were generated or analysed during the current study.

## References

[1] Tosetti I, Kuper H. Do people with disabilities experience disparities in cancer care? A systematic review. PLoS One. 2023;18(12):e0285146. 10.1371/journal.pone.0285146. PMID: 38091337; PMCID: PMC10718463. 10.1371/journal.pone.0285146 PMC10718463 38091337

[2] Adebisi YA, Ekpenyong A, Ntacyabukura B, Lowe M, Jimoh ND, Abdulkareem TO, Lucero-Prisno DE (2020). COVID-19 highlights the Need for Inclusive Responses to Public Health Emergencies in Africa. Am J Trop Med Hyg.

[3] Hempel M (1996). Reproductive health and rights: origins of and challenges to the ICPD agenda. Health Transition Rev.

[4] United Nations. Convention on the Rights of Persons with Disabilities (CRPD) | Division for Inclusive Social Development (DISD). 2006. https://legal.un.org/avl/pdf/ha/crpd/crpd_e.pdf. Accessed on June 17, 2024.

[5] The missing Billion. Access to health services for 1 billion people with disabilities. 2019. https://www.lshtm.ac.uk/media/38726. Accessed on June 17, 2024.

[6] Frawley P (2023). Access to sexual rights for all people with disabilities: the need to see and include the experiences of people with intellectual disability. Arch Sex Behav.

[7] Carew MT, Braathen SH, Swartz L, Hunt X, Rohleder P (2017). The sexual lives of people with disabilities within low- and middle-income countries: a scoping study of studies published in English. Glob Health Action.

[8] Groce NE, Rohleder P, Eide AH, MacLachlan M, Mall S, Swartz L (2013). HIV issues and people with disabilities: a review and agenda for research. Soc Sci Med.

[9] Groce NE. HIV/AIDS and individuals with disability. Health and Human rights., Koller TS, Thomas R, Manandhar M, Lustigova E, Diop A, Magar V. Tools and approaches to operationalise the commitment to equity, gender and human rights: towards leaving no one behind in the Sustainable Development Goals. Glob Health Action. 2018;11(sup1):1463657. 10.1080/16549716.2018.1463657. 10.1080/16549716.2018.1463657 PMC5974708 29808773

[10] World Health Organisation [WHO]: Health in 2015: from MDGs, millennium development goals to SDGs, sustainable development goals. 2015.

[11] Bolarinwa OA, Boikhutso T. Mapping evidence on predictors of adverse sexual and reproductive health outcomes among young women in South Africa: a scoping review. Afr J Primary Health Care & Family Med. 2021;13(1). 10.4102/phcfm.v13i1.3091 PMC8661283 34797120

[12] Löfgren-Mårtenson L (2012). I want to do it right! A pilot study of Swedish sex education and young people with intellectual disabilities. Sex Disabil.

[13] National Disability Insurance Scheme (NDIS). Inquiry talks about sexual expression in NDIS plans. 2023. https://www.sbs.com.au/news/podcast-episode/inquiry-talks-about-sexual-expression-in-ndis-plans/26o3vhepb. Accessed on: June 17, 2024.

